# Longitudinal effect of transcranial direct current stimulation on knee osteoarthritis patients measured by functional infrared spectroscopy: a pilot study

**DOI:** 10.1117/1.NPh.7.2.025004

**Published:** 2020-05-07

**Authors:** Luca Pollonini, Hongyu Miao, Hyochol Ahn

**Affiliations:** a University of Houston, Department of Engineering Technology, Houston, Texas, United States; b University of Houston, Department of Electrical and Computer Engineering, Houston, Texas, United States; c University of Texas Health Science Center at Houston, School of Public Health, Houston, Texas, United States; d University of Texas Health Science Center at Houston, Cizik School of Nursing, Houston, Texas, United States

**Keywords:** functional near-infrared spectroscopy, transcranial direct current stimulation, pain, knee osteoarthritis

## Abstract

**Significance:** Knee osteoarthritis (OA) is a common joint disease causing chronic pain and functional alterations (stiffness and swelling) in the elderly population. OA is currently treated pharmacologically with analgesics, although neuromodulation via transcranial direct current stimulation (tDCS) has recently generated a growing interest as a safe side-effect free treatment alternative or a complement to medications for chronic pain conditions. Although a number of studies have shown that tDCS has a beneficial effect on behavioral measures of pain, the mechanistic action of neuromodulation on pain sensitivity and coping at the central nervous system is not well understood.

**Aim:** We aimed at observing longitudinal changes of cortical hemodynamics in older adults with knee OA associated with a two-week-long tDCS self-treatment protocol.

**Approach:** Hemodynamics was measured bilaterally in the motor and somatosensory cortices with functional near-infrared spectroscopy (fNIRS) in response to thermal pain induced ipsilaterally to the knee primarily affected by OA.

**Results:** We found that both oxyhemoglobin- and deoxyhemoglobin-related functional activations significantly increased during the course of the tDCS treatment, supporting the notion that tDCS yields an increased cortical excitability. Concurrently, clinical measures of pain decreased with tDCS treatment, hinting at a potential spatial dissociation between cortically mediated pain perception and suppression and the prevalence of neuromodulatory effects over cortical pain processing.

**Conclusions:** fNIRS is a valid method for objectively tracking pain in an ambulatory setting and it could potentially be used to inform strategies for optimized tDCS treatment and to develop innovative tDCS protocols.

## 1 Introduction

Knee osteoarthritis (OA) is the most common type of joint disease in older adults, affecting more than 14 million people in the United States alone.^1^^–^^6^ OA manifests symptomatically in the form of chronic knee pain and disability, which are typically treated pharmacologically with analgesics. Recent studies have shown that pharmacological intervention alone is a suboptimal strategy for the management of knee OA in older adults since pain is only partially responsive and these drugs often produce significant adverse events, such as constipation, nausea, and drowsiness.^7^^–^^9^ Another challenge underscored by several studies is the poor correspondence between measures of OA disease severity and measures of clinical pain, hinting that pain processing at the central nervous level in the brain may play a significant role in addition to localized joint pathophysiology.^10^^,^^11^ In support of this theory, neuroimaging using functional magnetic resonance imaging (fMRI) have shown that pain-related brain activation in people with knee OA and alterations in pain-related brain mechanisms have been associated with OA-related clinical pain,^12^^–^^16^ possibly explaining the limited success of treatment locally targeting the pain in the area of the knee. Hence, there exists the need and the opportunity to investigate alternative and/or complementary strategies for pharmacological interventions for the treatment of OA-related pain.

Among innovative approaches to OA management, transcranial electrical stimulation (tES) has generated a growing interest as a potential treatment for chronic pain conditions. Although mechanisms of pain sensitivity, processing, and coping in the central nervous system are very complex and are still being widely investigated, a number of studies have shown that the neuromodulatory effect of electrical current on neuronal regions involved in pain processing had a benefical effect on behavioral measures of pain, thus supporting tES as a valid noninvasive therapeutic approach.^17^^–^^19^ The best-known form of electrical brain stimulation is transcranial direct stimulation (tDCS), consisting of a low-amplitude (2 mA or less) direct current applied to the head via two electrodes (anode and cathode) with the intent of modulating the resting membrane potentials of neurons located underneath and in-between electrodes, ultimately increasing the excitability of the targeted cortical area. Of relevance to clinical applicability, the U.S. Food and Drug Administration classifies tDCS (with its currently established dose of 2 mA max for 20  min/day) as a noninvasive, painless, and risk-free technique, which facilitates investigations and clinical trials for testing the safety and efficacy of tDCS.^20^^,^^21^ For chronic pain treatment, tDCS is typically delivered with the anode (i.e., the positively charged electrode) placed over the primary motor cortex (M1) of the hemisphere contralateral to the pain-affected area of the body, and the cathode (i.e., the negatively charged electrode) is placed over the supraorbital region (SO) ipsilateral to the affected area.^19^^,^^22^ In order to achieve clinically meaningful pain outcomes, tDCS is typically administered on a daily basis over the course of a few weeks.^23^ This M1-SO electrode montage purportedly increases the excitability of afferent or efferent neuronal structures involved in pain processing and increases pain inhibitory controls on the motor, somatosensory, and frontal cortices implicated in pain sensitivity.^24^^–^^29^ Our group^30^^–^^34^ as well as others^24^^,^^25^^,^^27^^–^^29^^,^^35^ have shown that a 2-mA current, M1-SO tDCS treatment applied in the clinic effectively improves chronic pain function in a variety of populations, including knee OA. In addition, of relevance to this study, the portability, connectivity, and relative ease-of-use of modern tDCS devices make this neuromodulation technique suitable for an in-home, self-administered regimen that avoids patients’ daily visits to a clinical site.^31^^,^^36^

In this paper, we present a pilot study designed to observe the longitudinal changes in the cortical hemodynamic response to thermal pain measured with functional near-infrared spectroscopy (fNIRS) concomitant to a two-week-long tDCS treatment protocol self-administered by older adults with knee OA, which, to our knowledge, is an unprecedented effort. We also collected measures of pain and OA-related symptoms to evaluate the therapeutic effect of tDCS treatment alongside hemodynamic changes. Although it is beyond the scope of this pilot study, cortical hemodynamics measured with fNIRS has the potential of enabling a better understanding of the underlying pain processing mechanisms at the central nervous level. Notably, the relationship between tDCS treatment and clinical measures of pain is undemonstrated, although our group has recently significantly contributed to this quest.^30^^–^^34^

Briefly, fNIRS is an optical technique that measures cerebral hemodynamic activity at the cortical level by illuminating the cortical layer with nonionizing near-infrared light (i.e., with wavelengths between 650 and 1000 nm) and by detecting the light back scattered by such tissues. fNIRS uses light at multiple wavelengths (typically two) to measure concentration changes of oxygenated hemoglobin (HbO) and deoxygenated hemoglobin (HbR), thus providing functionally relevant insight into cortical hemodynamics and oxidative metabolism.^37^ Although fNIRS can penetrate tissues no more than 3 cm underneath the scalp, it has excellent temporal resolution (>10  Hz) and sufficient spatial resolution at the cortical level (on the order of ${\mathrm{cm}}^{2}$) to investigate cortical hemodynamic activity through topographic imaging.^37^ In the past few years, several studies have demonstrated the feasibility, effectiveness, and practicality of using fNIRS for studying pain. Yucel et al.^38^ reported activations in the primary somatosensory cortex assessed with fNIRS in response to painful stimuli. Yennu et al.^39^ reported significant hemodynamic activity in the prefrontal cortex measured by fNIRS in response to thermally induced pain as previously revealed by pain-related fMRI studies. However, no studies to date have longitudinally investigated the cortical hemodynamic response to pain with the goal of understanding how tDCS affects pain-related brain activity among older adults with knee OA.

In this pilot study, we leveraged neuroimaging by fNIRS to increase our mechanistic knowledge underlying the analgesic effects of tDCS. Further, we evaluated fNIRS findings concomitantly with clinical measures of pain to enhance understanding of differences in tDCS responses. We found that both oxyhemoglobin- and deoxyhemoglobin-related functional activations significantly increased during the tDCS treatment, supporting the notion that tDCS yields an increase cortical excitability. We also found that cortical hemodynamic activity and clinical measures were inversely related over time, hinting at a potential spatial dissociation between cortically mediated pain perception and suppression, and at the prevalence of neuromodulatory effects over cortical pain processing.

In the long term, we hope that fNIRS neuroimaging in the ambulatory setting could be used to inform strategies for optimized tDCS treatment and to develop innovative tDCS protocols.

## 2 Materials and Methods

### 2.1 Subjects

For this pilot study, we recruited 10 individuals (9 females, 1 male, 62.4±6.9  years, OA-related pain suffering 37.7±31.5  months) affected by right knee OA from the greater Houston community. Consistent with our previous work,^30^ we included 50- to 85-year old participants with (1) symptomatic knee OA based on American College of Rheumatology Clinical criteria,^40^ (2) recent experience of knee OA pain, i.e., a self-reported score of 30 or greater (out of 100) on a visual analog scale (VAS)^41^ for pain within the past 3 months, (3) ability to speak and read English, (4) access to an internet-connected device for real-time remote supervision via secure videoconferencing, and (5) no planned change in the medication pain regimen.

Participants with any concurrent medical conditions that could confound interpretation of outcome measures, posed a safety risk for any of the assessment or tDCS procedures, or precluded successful completion of the protocol were excluded from the study. More specifically, exclusion criteria were: (1) prosthetic knee replacement or nonarthroscopic surgery on the affected knee; (2) history of brain surgery, brain tumor, seizure, stroke, or intracranial metal implantation; (3) systemic rheumatic disorders, including rheumatoid arthritis, systemic lupus erythematosus, and fibromyalgia; (4) alcohol/substance abuse or diminished cognitive function; (5) pregnancy or lactation; and (6) hospitalization within the preceding year for psychiatric illness. The experimental protocol was approved by the Institutional Review Board of the University of Texas Health Science Center.

### 2.2 Experimental Protocol

Upon recruitment and consenting to the experimental protocol, participants were trained in the use of portable tDCS equipment (1×1 tDCS, Soterix Inc., NY) and were asked to self-administer a 20-min long, 2-mA dose of tDCS every weekday (Monday to Friday) for two weeks (i.e., for a total of 10 tDCS sessions) at home or in a private room in which participants felt comfortable. The tDCS device included two $5\times 7\;{\mathrm{cm}}^{2}$, saline-soaked sponge electrodes, acting as anode and cathode, respectively, placed with the aid of a headgear on the primary motor cortex M1 of the left cerebral hemisphere (C3 according to the international 10 to 20 measurement system, contralateral to the painful knee) and on the supraorbital area SO of the right cerebral hemisphere (Fp2 according to the international 10 to 20 measurement system, ipsilateral to the painful knee). The direct current intensity (2 mA) and duration (20 min) were preset without possibility of being altered by the participant. tDCS self-administration was remotely supervised by our research staff via a secure video conference system (WebEX provided by UTHealth Communications Technology and Interactive Video Services) to ensure safety and compliance with the experimental protocol. All participants complied with the 10-day, 20-min-per-day tDCS protocol and no one reported adverse side effects.

Clinical pain was measured with the VAS for pain, i.e., a self-reported estimation of knee pain perception on a 0 to 100 scale,^30^^,^^41^ and OA symptoms were measured with the Western Ontario and McMaster Universities Osteoarthritis Index (WOMAC),^42^ i.e., an OA-specific cumulative index ranging from 0 to 96 and resulting from the summation of quantitative assessments of pain, stiffness, and function of the knee. Both VAS and WOMAC measures were collected at baseline (i.e., day 0, prior to the first tDCS session) and weekly (i.e., days 5 and 10) immediately after the administration of the 20-min long tDCS treatment. Data collection was conducted at similar times on all experimental days, allowing only slight variation due to the subjects’ daily schedules. Critically, subjects were inquired at all sessions about the intake of prescribed or over-the-counter analgesic medications, but none of the subjects reported any OA-specific pharmacological intervention. During the baseline visit, we also collected data on heat pain sensitivity using an ascending method of limits. Using a temperature-controlled, $16\times 16\;{\mathrm{mm}}^{2}$ thermode (Medoc TSA-II Neurosensory Analyzer) applied to the right forearm ipsilateral to the OA-affected right knee, the temperature was initially set at 32°C and increased at a rate of 0.5°C/s until the participants pressed a button to indicate when their heat perception became painful. On average, the pain threshold temperature was found to be 37.90°C±3.30°C (mean±standard deviation).

**Fig. 1:**
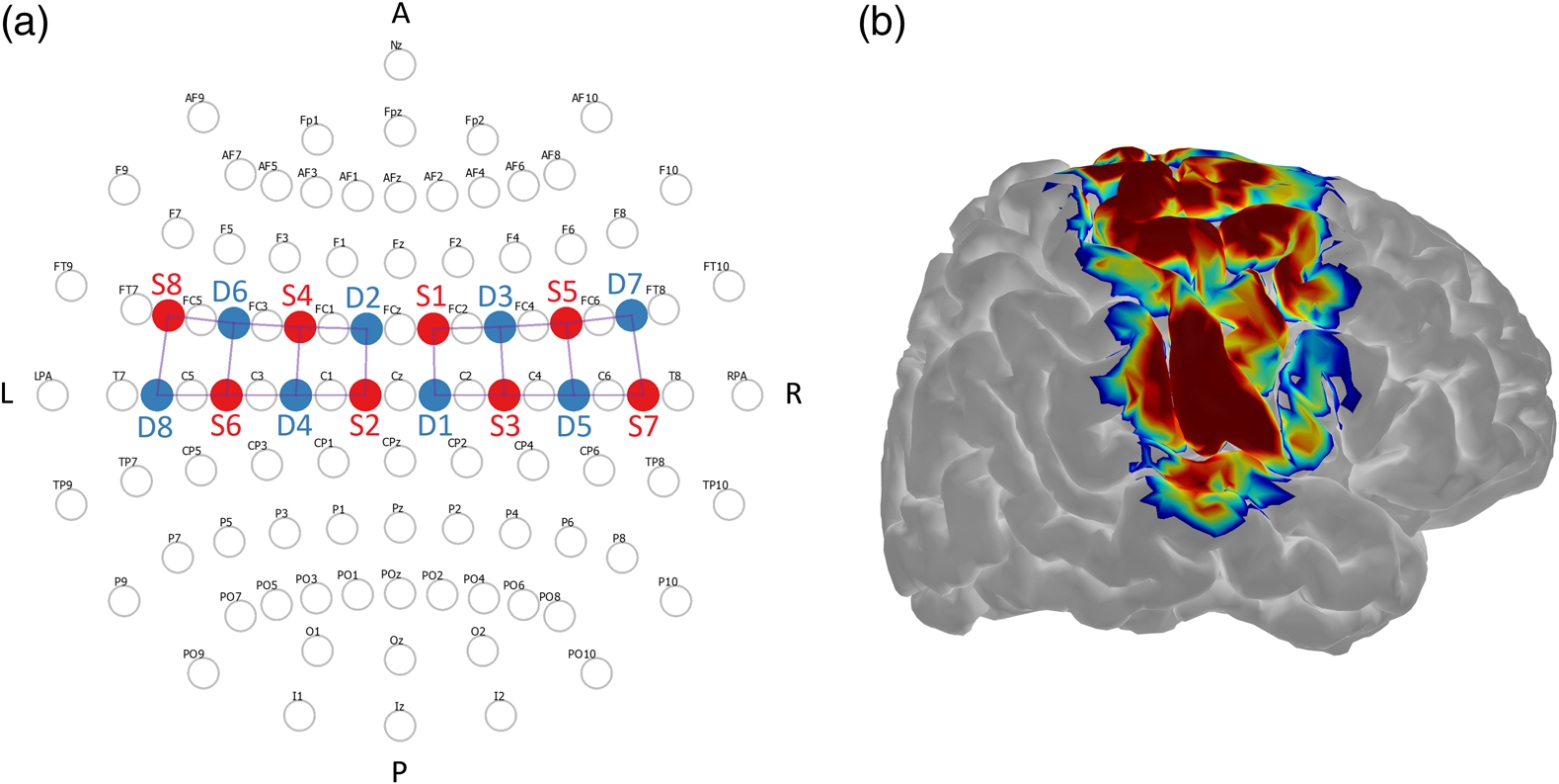
(a) Geometrical layout of sources (S, red) and detectors (D, blue) with respect to the international 10–10 electroencephalogram (EEG) system,^44^ and (b) correspondent sensitivity map of the right hemisphere (symmetric to the left hemisphere sensitivity map) overlaid onto the Colin27 brain model. Sensitivity computed and displayed with AtlasViewer.^45^

Pain-related cortical response was measured using a continuous-wave fNIRS imaging system (LIGHTNIRS, Shimadzu, Kyoto, Japan). The instrument encompassed eight light sources (each consisting of three semiconductor lasers at 780, 805, and 830 nm) and eight detectors connected by optical fibers to a head-fitting elastomeric headgear. The illumination and detection optodes were arranged in a grid-like layout covering the primary motor and somatosensory cortices bilaterally (Fig. 1, Ref. 43), consistent with cortical locations investigated in previous studies.^38^^,^^46^^–^^48^ The optodes arrangement yielded a total of 20 optical channels (i.e., 10 per hemisphere) in which the source–detector interdistance was nominally set at 30 mm with the help of linked optode holders.

**Fig. 2:**
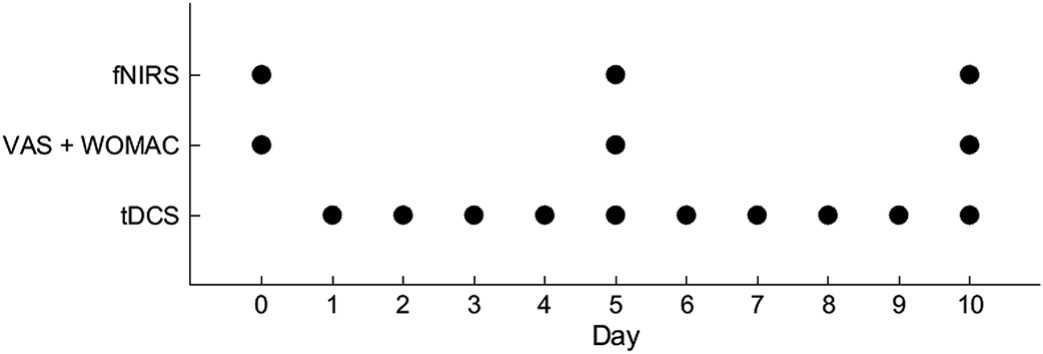
Timeline of the experimental protocol.

Optical readings in each optical channel were collected at a frequency of 13.3 Hz during thermal pain stimulation, which was produced in a block design paradigm onto the right forearm aimed at reproducing OA-induced lateralized pain. After an initial baseline period of 60 s during which no thermal stimulation was applied (i.e., thermode at room temperature), the thermode temperature had risen to 45°C (i.e., supra heat pain threshold) to induce a moderate-to-intense sensation of pain for 20 s. After the active stimulation, a 30-s recovery period with no thermal stimulation followed. The experimental block was repeated six times for a total of 390 s that included a 60-s resting period at the end of the stimulation chain (i.e., 60 s baseline +6×20  s thermal stimulation +5×30  s thermal recovery between thermal stimulations +60  s at the end of stimulation). Each experimental session lasted about 20 to 25 min, including the subject preparation time. Concomitantly with the clinical assessment of pain, fNIRS data were obtained at 3 points in time: at baseline (prior to the tDCS regimen) and for each of the two weeks thereafter (i.e., day 5 and day 10 of tDCS treatment). The entire experimental timeline is summarized in Fig. 2.

### 2.3 Data Analysis

**Fig. 3:**
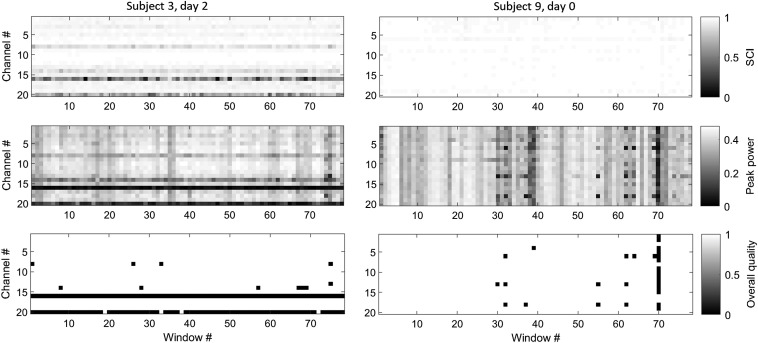
Image plots of SCI (top row), peak power (middle row), and overall quality (0=SCI<0.8 and peak<0.10) for two selected fNIRS scans (left and right columns). On the left fNIRS scan (subject 3 on day 2), channels 16 and 20 have low quality due to poor contact with the scalp throughout the experiment. On the right scan (subject 9 on day 0), the quality was overall sufficient with the exception of time window #70 where most channels were degraded by a motion artifact.

Raw optical data were initially evaluated for quality based on an approach combining an automatic detection of problematic signals and visual inspection. To automatically detect low-quality recordings, we applied a method introduced in our previous work that quantified the optical coupling between the optodes of an optical channel and the scalp from the strength of the cardiac pulsation present in each optical signal.^49^^–^^51^ More specifically, we partitioned optical signals from each optical channel into 5-s time windows, bandpass filtered (0.5 to 2.5 Hz), and normalized the signals at both wavelengths to preserve only the cardiac oscillation and computed (a) the scalp contact index (SCI), i.e., a 0 to1 score (1 being the best) as the zero-lagged cross correlation between the signals and (b) the peak power of such a cross correlation. In essence, a good optical coupling between optodes and scalp yields raw optical signals with a prominent cardiac pulsation, which, in turn, yields high values of SCI and peak power measures. For each optical channel, each 5-s signal window was classified as of high quality if the scalp coupling index was >0.8 or the peak power was >0.1. In our previous work,^51^ we showed that this methodology accurately detects experiment-long noisy optical signals (mostly attributed to poor scalp−optode coupling) as well as spurious, temporary movement artifacts. We hereby report two examples of fNIRS scans containing optical channels with experiment-long poor scalp contact (Fig. 3, left column) and a single-time window affected by a motion artifact (Fig. 3, right column). Notably, the signal processing applied to assess data quality was used merely to identify noisy scans to be discarded and it was not carried over to the subsequent analysis of clean recordings. To confirm the sufficient quality of the remaining recordings, we also visually inspected the signals to detect obviously problematic signals that would have required exclusion. Ultimately, one subject out of 10 had to be discarded due to excessive noise found in all three recording sessions.

Subsequent to quality assessment of the optical signals, raw data were converted to optical density and then to changes of concentration of HbO and HbR over time according to the modified Beer–Lambert law.^52^^,^^53^ Individual-level and group-level analyses of cortical hemodynamic readings are described further in Sec. 2.4. All data quality assessment algorithms were developed by our group, whereas conversion from raw data to hemodynamic parameters and individual and group-level analyses were performed using the software AnalyzIR.^54^ All code was executed in MATLAB® (Natwick, MA).

### 2.4 Statistical Analysis

Clinical pain and OA-symptoms measurements (VAS and WOMAC) were analyzed statistically with repeated measures analysis of variance (ANOVA) (within-subject levels: days 0, 5, and 10, significance level 5%, multiple comparisons corrected with Tukey’s method) to evaluate the effect of the tDCS treatment over the course of the 2-week protocol.

The relationship between cortical hemodynamic activity (i.e., changes in HbO and HbR over time) and thermal stimulation was quantified at the subject level (i.e., each optical channel of each subject) with a general linear model (GLM) based on the autoregressive iteratively reweighted least squares (AR-IWLS) approach described in Ref. 42, where only optical channels with p values lower than 5% were considered significantly active (i.e., beta weight statistically different from zero). We hypothesized a canonical time profile of the hemodynamic response to thermally induced pain, i.e., a double gamma function.^54^ Notably, the AR-IWLS method automatically accounts for the expected presence of extracerebral components in the optical signals due to cardiac pulsation and respiration. Hence, a bandpass signal filtering step was not explicitly required to extract cerebral-only hemodynamics. At the group level, we assessed the relationship between the estimated beta coefficient of each optical channel (the predicted variable) and the effect of time (experimental days 0, 5, and 10) using a linear mixed-effects model with the subject-specific intercept set as a random effect.

## 3 Results

### 3.1 Clinical Pain and OA Symptoms Measures

**Fig. 4:**
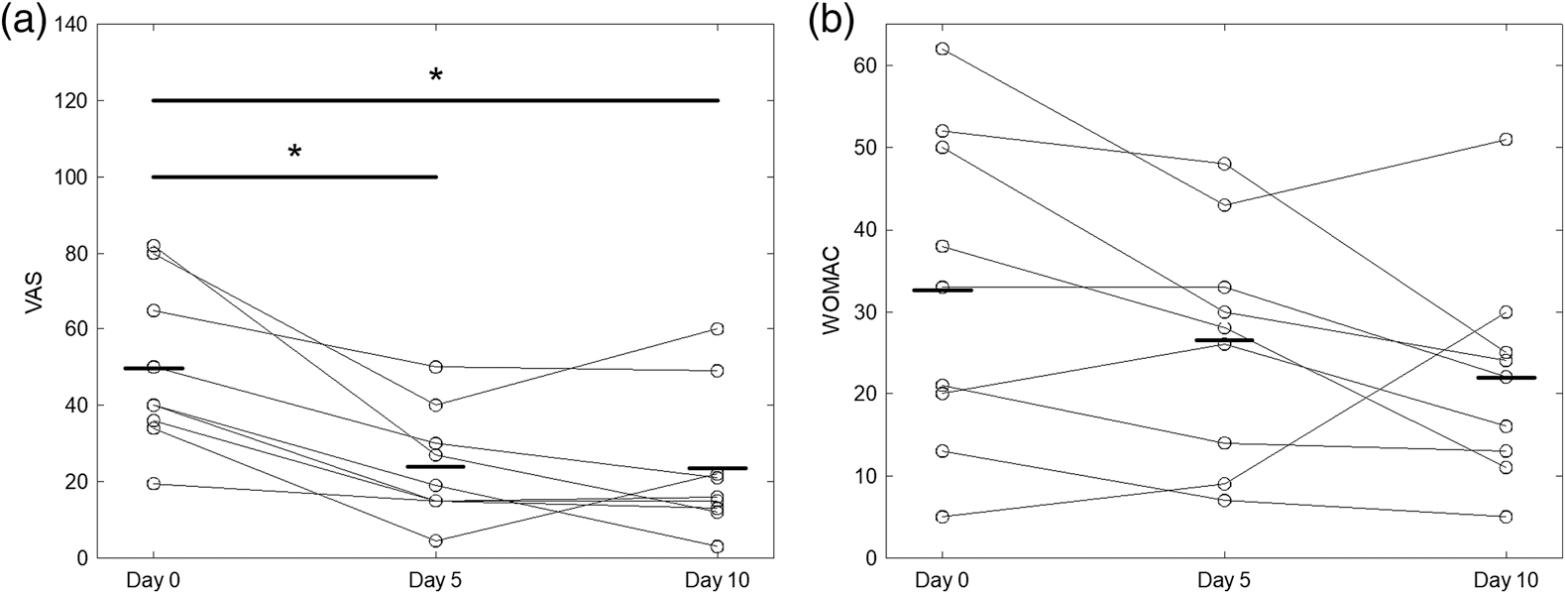
(a) Individual VAS and (b) WOMAC scores for each session, along with mean value (bold line) and statistically significant comparisons (*p<0.05).

Both VAS and WOMAC measures decreased over the 2-week course of treatment (Fig. 4). Time was found to be a statistically significant factor with respect to VAS ($p_{\mathrm{VAS}}=0.0001$), but not to WOMAC ($p_{\mathrm{WOMAC}}=0.07$). VAS at corresponding weekly measures was significantly lower than baseline VAS ($p_{\mathrm{day}\;5}=0.002$, $p_{\mathrm{day}\;10}=0.008$). However, the difference between VAS scores on days 5 and 10 was not significant (p=0.99). Likewise, WOMAC scores were not significantly different when taken pairwise (p>0.16).

### 3.2 Optical Neuroimaging

**Fig. 5:**
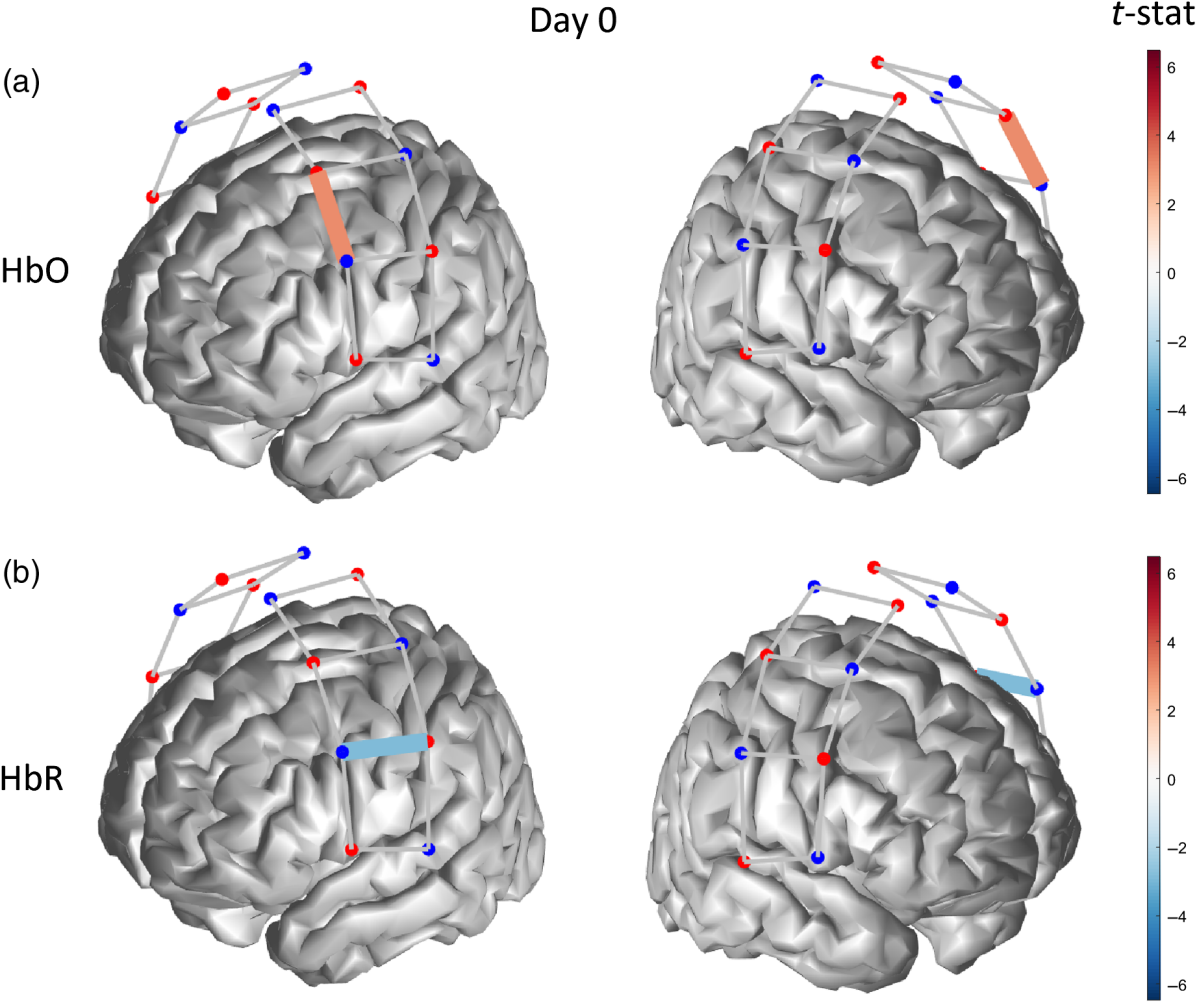
Functionally active optical channel (colored links) for (a) HbO and (b) HbR in response to thermal stimulation on the forearm before the tDCS treatment.

Before starting the tDCS treatment, subjects exhibited a single-channel activation in the left hemisphere in response to thermal pain, thus contralateral to the stimulation site (right forearm) for both HbO (p=0.028) and HbR (p=0.038) (Fig. 5). For clarity, we define an activation as an increase of HbO toward positive values or a decrease of HbR toward negative values, whereas we refer to a deactivation as a decrease of HbO toward negative values or an increase of HbR toward positive values. This is consistent with the generally accepted notion that a neuronal activation in response to stimulation produces an increase of HbO concentration and a corresponding decrease in HbR concentration.

**Fig. 6:**
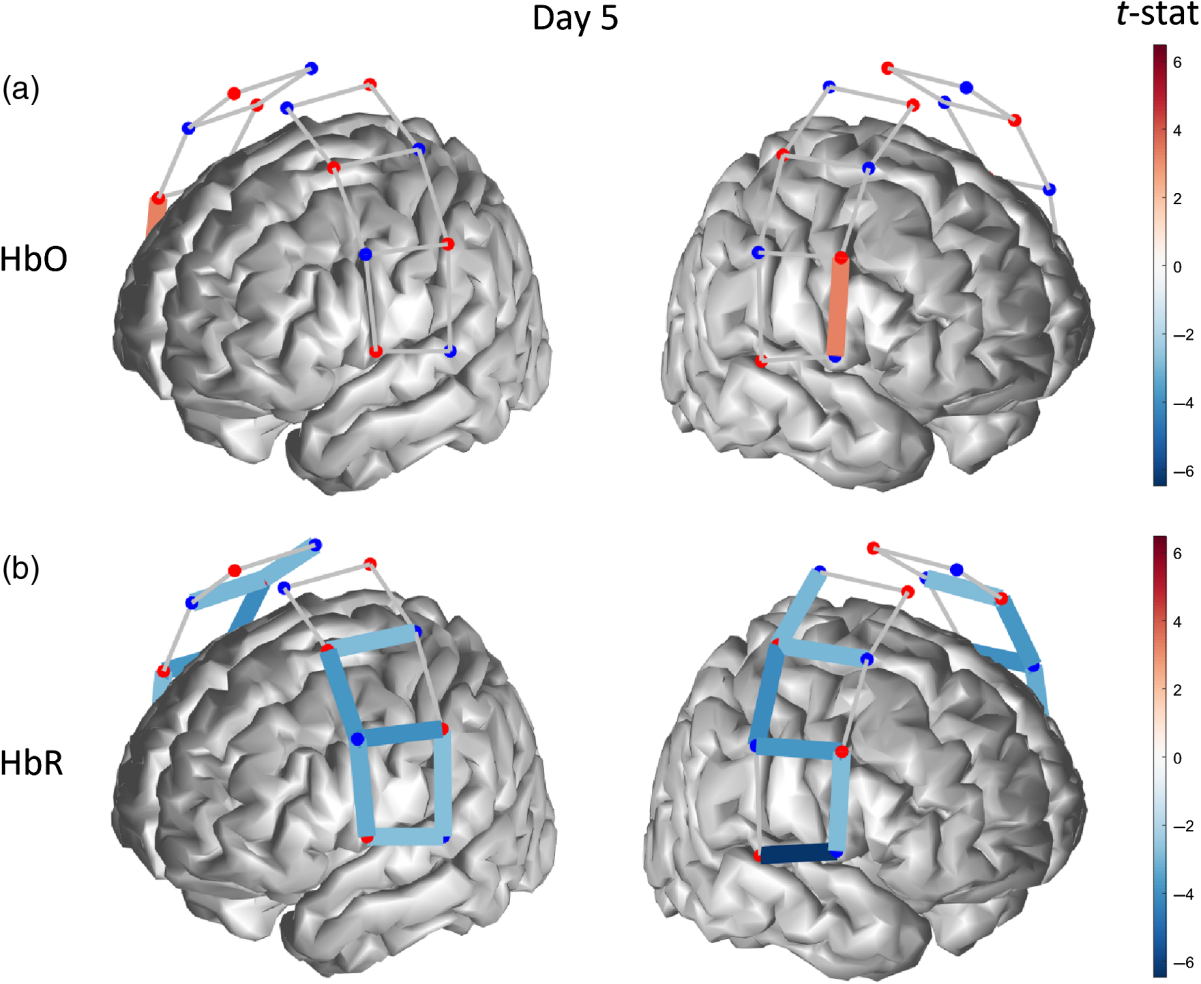
Functionally active optical channel (colored links) for (a) HbO and (b) HbR in response to thermal stimulation on the forearm after 5 days of tDCS treatment.

After five consecutive days of self-administered tDCS treatment, the cortical hemodynamic response to thermal stimulation registered a single-channel activation in the anterior–inferior right hemisphere for HbO (p=0.022) and a widespread bilateral activation for HbR (p<0.036 for all active channels) (Fig. 6).

**Fig. 7:**
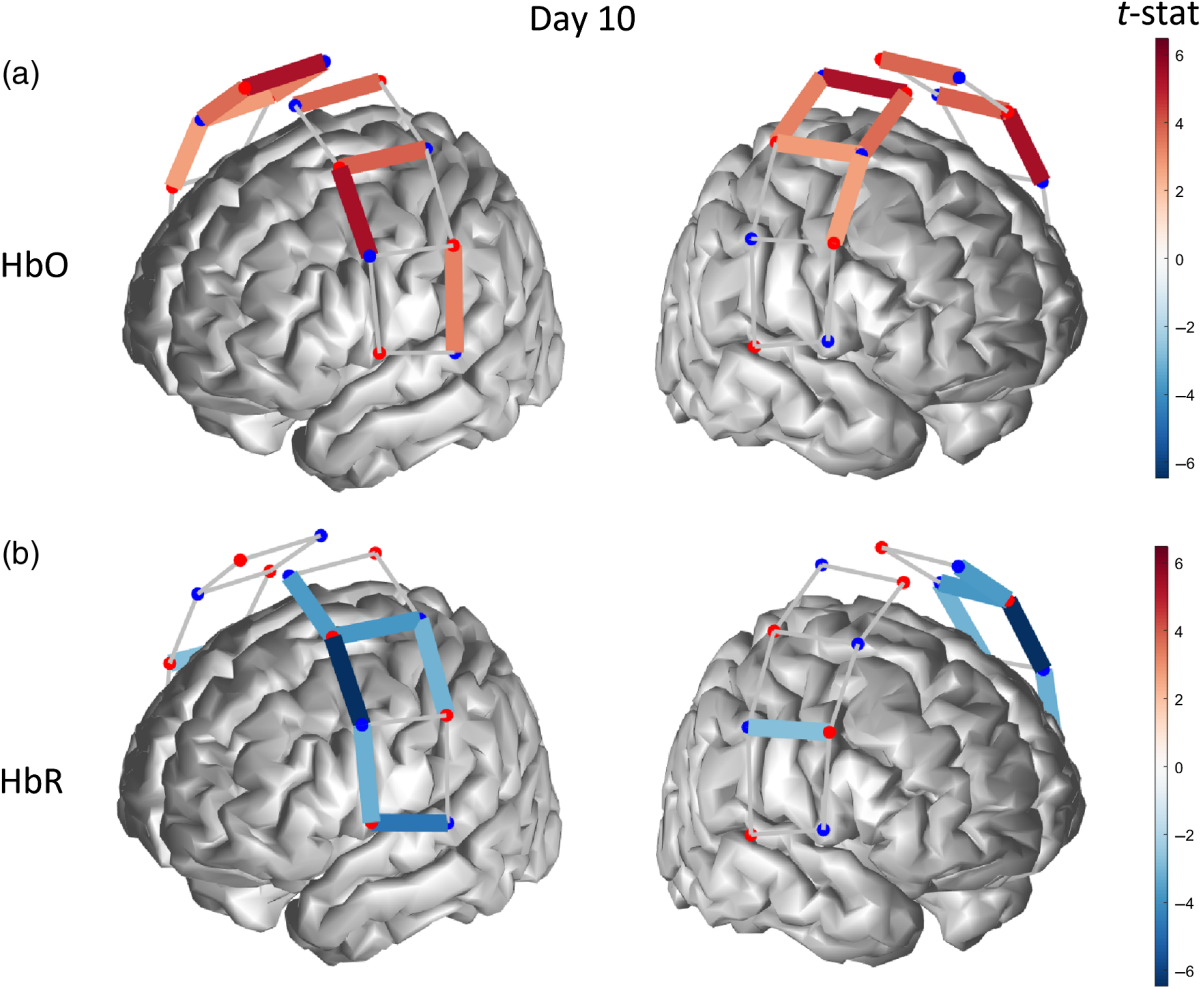
Functionally active optical channel (colored links) for (a) HbO and (b) HbR in response to thermal stimulation on the forearm after 10 days of tDCS treatment.

At the conclusion of the 10-day tDCS treatment, we measured a significant bilateral hemodynamic activation (p≤0.042) for HbO in middle-superior regions of the probed cortex [Fig. 7(a)], accompanied by an activation pattern for HbR (p≤0.022) spanning most channels of the left hemisphere, i.e., contralateral to the thermal stimulation and a single-channel activation in the right hemisphere [Fig. 7(b)].

**Fig. 8:**
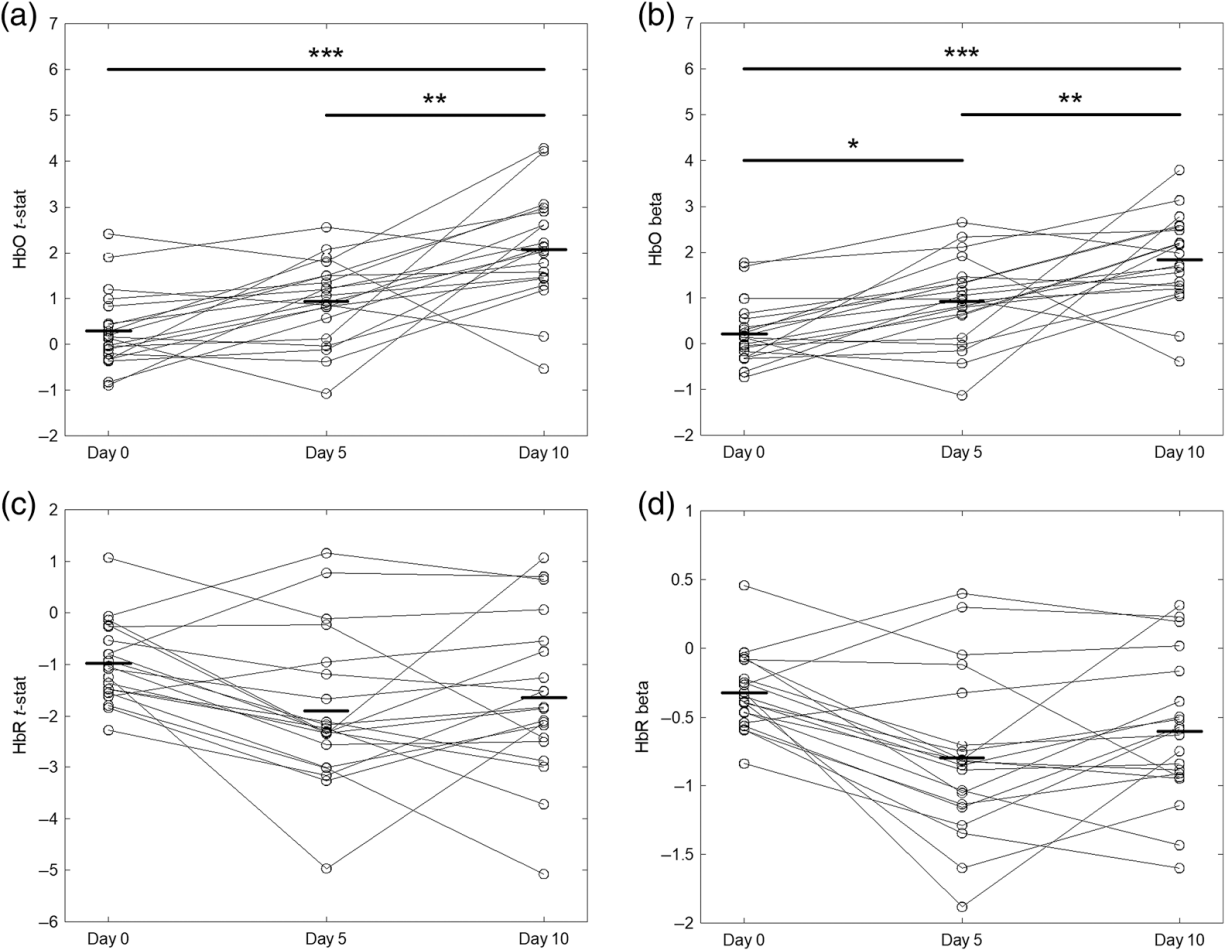
(a), (c) T-statistics and (b), (d) beta weights for (a), (b) HbO and (c), (d) HbR of each optical channel, along with mean values (bold line) and statistically significant comparisons. (*p<0.05, **p<0.005, ***p<0.0005).

Differences between the hemodynamic maps across different tDCS treatment days were tested for significance using ANOVA corrected for multiple comparison with Tukey’s method. Figures 8(a) and 8(c) show channel-level t-statistics of HbO and HbR (representing the significance of the hemodynamic response to thermal stimulation) as a function of the measurement day. We found that the level of HbO activity measured on day 10 (last day of tDCS treatment) was significantly larger than the related activity on both day 0 (p<0.0000) and day 5 (p<0.0013), whereas HbR activity was not significantly different across the three measurement days. Beta weights representing the amplitude of the hemodynamic response to thermally induced pain exhibited trends similar to the t-statistics for both HbO and HbR, although we also found that the difference between HbO activities at day 0 and day 5 was statistically significant [p=0.035, Fig. 7(b)].

**Fig. 9:**
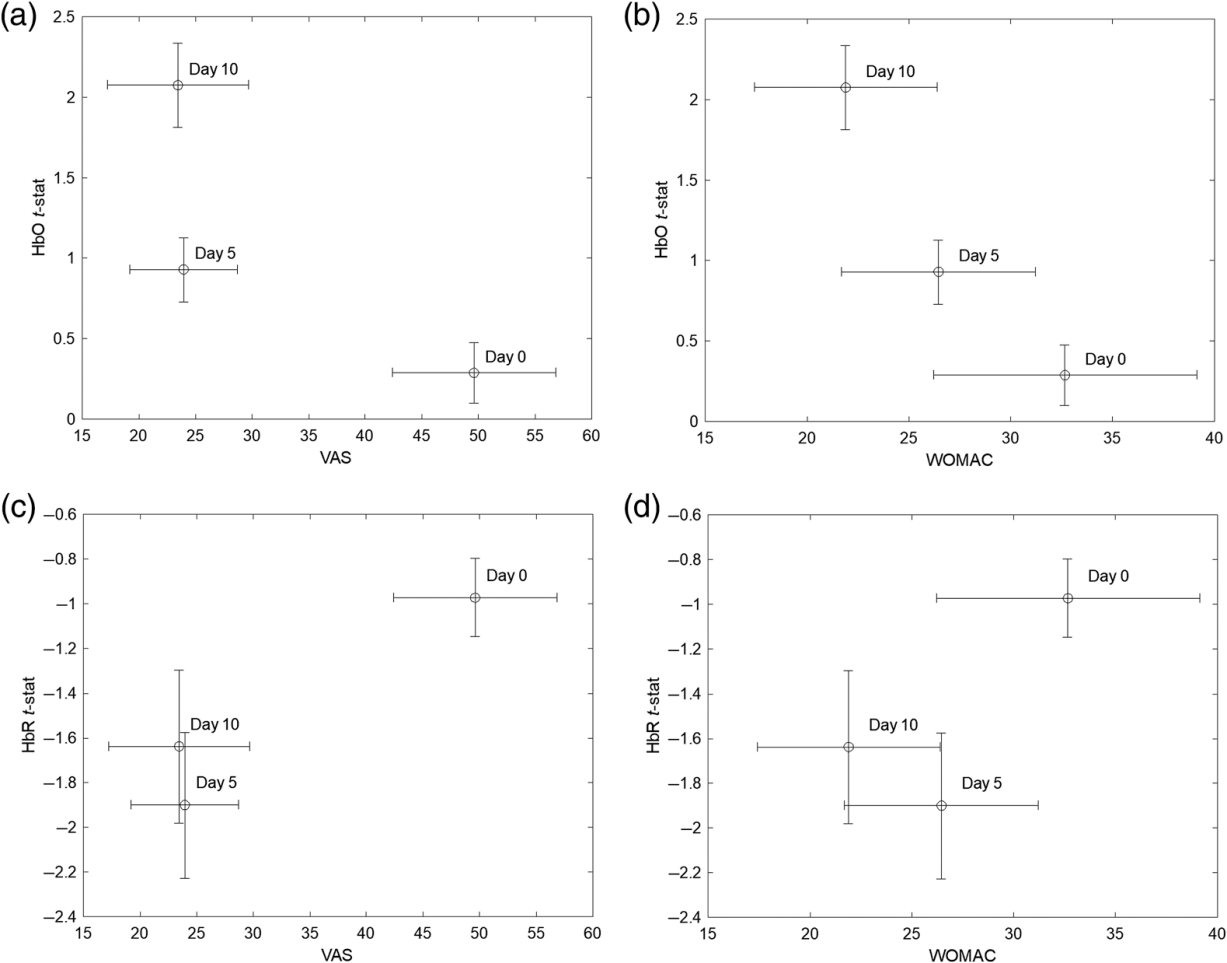
Clinical measures VAS: (a), (c); WOMAC: (b), (d) and hemodynamic activity measures HbO: (a), (b); HbR: (c), (d) for the three experimental sessions (day 0, day 5, and day 10). Mean (circle marker) and standard error (error bars).

Finally, a summary of clinical pain and OA symptom measures (VAS and WOMAC) and hemodynamic activity (t-statistics for HbO and HbR) for all three experimental days is shown in Fig. 9. As visually anticipated by the hemodynamic maps presented above, cortical hemodynamic activities changed inversely with respect to clinical measures collected during the 10-day tDCS treatment. Specifically, the t-statistics for HbO increased from day 0 to day 10 in association with a decrease in perceived pain (VAS) and functional pain (WOMAC) [Figs. 9(a) and 9(b)]. Consistently, the HbR cortical activity also increased (i.e., toward more negative values) in association with a decrease of both pain measures [Figs. 9(c) and 9(d)] as the tDCS treatment progressed, although we noted the above HbR changes were not significantly different [Figs. 8(c) and 8(d)]. Both HbO and HbR did not exhibit significant changes from day 0 to day 5 despite a significant decrease in VAS pain perception (WOMAC also did not decrease significantly, Fig. 4). In contrast, the HbO change from day 5 to day 10 was statistically significant, whereas the VAS pain perception was not. However, both HbO and VAS levels were significantly different between day 0 and day 10.

## 4 Discussion

In this pilot study, we aimed at observing the longitudinal effect of a two-week tDCS treatment on the cortical hemodynamics measured by fNIRS of a knee OA cohort. To our knowledge, this is the first investigation to observe changes in pain-related cortical response using fNIRS following self-administered tDCS in older adults with knee OA. Notably, our group is currently investigating the efficacy of tDCS as a therapeutic, nonpharmacological approach to chronic knee pain in a series of randomized clinical trials, and pilots studies conducted thus far have shown a significant reduction of pain perception associated with neuromodulation.^30^^,^^31^^,^^31^^–^^34^ A large-scale demonstration of the effects of tDCS on clinical pain through a clinical trial has yet to be completed, hence the goals of this pilot study of relating fNIRS-derived cortical hemodynamics to longitudinal tDCS and to self-reported pain measures (regardless whether the pain is altered by tDCS alone or in addition to other factors) can be considered independent from each other. Also of relevance, prior research had used fNIRS to measure the cortical hemodynamic response to thermal pain,^39^^,^^46^^,^^47^^,^^55^ but our pilot study appears to be the first where fNIRS data in response to thermal stimulation has been acquired concurrently with a tDCS treatment longitudinally for two weeks (three longitudinal sessions: one observation before treatment and two observations during the treatment).

### 4.1 Effect of tDCS on Clinical Measures

As in our previous studies on knee OA populations,^30^^,^^31^ we utilized thermal stimulation on the forearm ipsilateral to the index knee (i.e., the knee with dominant pain) as an experimental paradigm to induce noxious pain. This approach is commonly used in many lab-based experimental pain studies.^32^^,^^56^ We found that both general pain measure (VAS) and OA-symptom measure (WOMAC) decreased over the course of the two weeks of tDCS treatment, thus confirming the beneficial effect of neuromodulation by tDCS on a knee OA population as demonstrated in our previous research conducted in the clinical setting^30^ as well as in the home setting.^31^ Although the present pilot study involved a smaller population sample (N=9) and had no sham-tDCS condition, the self-administration of tDCS achieved the desired effects of a reduced pain perception, which consistently supports the possibility for use on a much larger population of patients in both research and clinical scenarios. Critical for training and safety, our group video-surveilled the OA patients remotely during tDCS self-administration and all participants tolerated home-based tDCS well without experiencing any serious adverse effects.

### 4.2 Effect of tDCS on Cortical Hemodynamics

One aim of the current pilot study was to assess whether or not optical neuroimaging acquired longitudinally using fNIRS captured changes induced by tDCS self-administered at a standard dose of 2 mA for 20  min/day for two weeks (5  days/week) using an M1(anode)-SO (cathode) layout. Our results show that the cortical hemodynamic activity associated with both HbO and HbR had an increasing trend from baseline (prior to the start of the protocol) to the end of the tDCS treatment (two weeks after), although only differences in HbO levels were statistically significant (see Fig. 8). When the statistical effect (t-statistics) of all 20 channels was pooled together to yield a comprehensive measure of hemodynamic activity across the whole optical probe, we determined that the cumulative HbO response to thermal stimulation had a strongly significant difference between baseline and end of treatment, but the second week of treatment alone induced a significant HbO increase (Fig. 8). Although most significantly active channels were contralateral to the stimulation site, we also detected a fairly large bilateral activity for HbO (day 10) and HbR (day 5). Bilateral cortical activation of the somatosensory cortex in response to thermal stimulation has also been shown in previous research, although the majority of the studies only considered HbO responses and only a few also reported the HbR activity.^46^

Our results indicate that tDCS induced hemodynamic changes of the probed cortex during the treatment period, but such changes became significant only after a prolonged tDCS regimen (i.e., two weeks in this study). In our view, this finding is consistent with the notion that neuromodulation attains a significant cortical excitability effect only when built cumulatively upon days of consecutive application,^57^^–^^59^ although a “target” prescription for tDCS is still widely investigated and, in all likelihood, it depends on the daily dosage (i.e., current duration and amplitude), electrodes placement, and, in the case of therapeutic use, the condition being treated.

Although there exist growing research literature exploring the effects of tDCS on cortical hemodynamic changes measured with fNIRS (see Ref. 60 for a comprehensive review of literature and applications), to the best of our ability, we could not find any study focused on the somatosensory cortex. Only a few studies, notably Khan et al.,^61^ Muthalib et al.,^62^ and Besson et al.,^63^ probed the sensorimotor cortex to investigate the effect of tDCS on motor tasks. In addition, no research seems to have looked at the longitudinal effects of tDCS on cortical hemodynamics beyond a single, short-timed tDCS application. Since our pilot study is also the first aimed at investigating a specific condition (knee OA) with fNIRS, we also have no prior research on this population with which we could compare our results.

Notably, the majority of fNIRS studies in pain research reported activations of HbO only due to excessive noise of the HbR signal. In contrast, we could measure significant HbR activity in response to thermal pain, especially after 5 and 10 tDCS sessions, and the longitudinally increasing trend of hemodynamic HbR activity was consistent with that of HbO. These findings support investigating HbR hemodynamics in spite of the typically weaker optical signals, especially in consideration of the notion that the HbR response is less affected by systemic components (i.e., scalp hemodynamics of particular relevance to pain) than the HbO response.^64^^–^^66^

In essence, with regards to the effect of tDCS on cortical hemodynamics, fNIRS represents a valid technique to investigate longitudinal changes induced by neuromodulatory techniques. This technique holds great potential for improving the understanding of the underlying mechanisms of transcranial electric stimulation on cortical excitability. As well-described by McKendrick,^60^ fNIRS comprises critical advantages compared to other neuroimaging techniques, including portability for use in ambulatory settings and compatibility with tES devices that allow investigating both online and offline effects of tES.

### 4.3 Concurrent Changes of Clinical Measures and Cortical Hemodynamics

Since neuroimaging, including but not limited to fNIRS, holds potential for providing innovative, objective measures of pain to complement self-reported yet clinically accepted pain assessment, we observed how thermally induced cortical hemodynamics changed alongside measures of pain (VAS) and OA symptoms (WOMAC) during the two-week tDCS regimen. Although the small sample size of this study did not allow to establish a statistically valid relationship, we found that both HbO and HbR cortical responses increased longitudinally, while clinical measures of OA-related pain and symptoms decreased.

Prior research has promisingly investigated the predictive value and repeatability of neuroimaging (mainly fMRI-based) measures of pain evoked thermally in healthy individuals.^67^^–^^69^ Previous studies commonly found that the blood oxygen level dependent (BOLD) response correlated positively with heat ratings (e.g., innocuous versus mildly noxious versus intensely noxious), but significantly less with pain measures like VAS.^69^ In addition, the response measured in the primary somatosensory cortex was found to be a less reliable indicator of pain level compared to other regions of interest (i.e., anterior and posterior insula and anterior cingulate cortex^67^) and that the biphasic hemodynamic response of the somatosensory cortex related to the heat intensity only in its late occurrence.^67^^,^^69^ These fMRI findings indicate that quantitative pain assessment via neuroimaging (fMRI specifically) need to account for both the spatially and the temporally distributed nature of pain processing, making it difficult to conclude whether our fNIRS findings are, in fact, supported by other neuroimaging modalities.

Thermal pain has been investigated also with fNIRS, albeit studies involving motor and somatosensory cortices are quite limited. Consistently with previous research,^38^^,^^47^ we measured bilateral activations for both HbO and HbR in response to noxious stimuli (Figs. 6 and 7), although our analysis assumed a canonical hemodynamic response function and did not analyze the specific time profile of the response. Another element of difference is that our longitudinal experiment did not include innocuous stimuli (e.g., a low-temperature condition), so we could not contrast hemodynamic activations across different pain intensity levels. Moreover, the thermal stimulation level (45°C) was kept consistent across subjects and sessions, although there exists the possibility that participants could have perceived pain differently (e.g., moderate versus intense pain). Due to the limited number of existing studies and experimental differences among them, it is evident that more research is needed to establish congruency between fNIRS results, especially in relation to clinical pain measures. Explaining the inverse longitudinal variation of pain levels and cortical hemodynamic activity also remains challenging due to the early stage of this research, although it is plausible that a spatial dissociation between pain perception and pain suppression mechanisms may exist, along with cognitive modulation processes that were not investigated in this pilot study. In addition, neuromodulatory and pain perception processes may be competing in terms of recruitment of the somatosensory cortex, with results indicating a longitudinal prevalence of the former.

### 4.4 Study Limitations and Future Directions

This pilot study arguably suffered from several limitations that will be addressed in our future research.

First, the limited number of optodes of our equipment (eight sources and eight detectors) allowed us to probe only the motor and somatosensory cortices, albeit bilaterally, with reasonable coverage and spatial resolution. Considering that several fNIRS studies have recently shown a significant role of the prefrontal cortex in the processing of thermal pain,^39^ we are currently conducting additional research in which both somatosensory and prefrontal cortices are interrogated. In the future, we believe that an even larger coverage would be able to better describe the complex, distributed mechanisms of pain processing across several cortical areas.

Second, our methodology did not encompass techniques for the reduction of the scalp hemodynamics^65^^,^^66^^,^^70^^,^^71^ that noxious stimuli may have added onto the targeted cortical hemodynamics. In this pilot study, we had opted to use all available optodes for probing a larger cortical area, but we may revise this trade-off in future investigation in favor of short-separation techniques. Third, although fitting the measured optical response with a predicted temporal function is a standard procedure for fNIRS analysis based on the GLM approach and has been used to investigate pain,^39^ other investigations^38^^,^^55^^,^^72^^,^^73^ indicated that the time profile of the hemodynamic response may have features that are unique to pain stimulation (thermal or otherwise). Since one primary goal of this pilot study was to longitudinally investigate hemodynamic changes on a limited number of subjects, observing the statistical measures derived from the GLM approach based on a canonical hemodynamic response allowed us to do so with sufficient statistical power. However, we consider it equally interesting to look at how temporal features may vary (or remain unaltered) as a function of time and/or analgesic interventions.

Finally, this pilot study did not include a sham-tDCS condition, as the primary goal was to observe longitudinal, concurrent changes of pain perception and hemodynamic response to thermal pain in a cohort undergoing an active tDCS treatment, rather than evaluating the therapeutic efficacy of tDCS and observing potential placebo effects.
